# Anti-Candidal Activity of Genetically Engineered Histatin Variants with Multiple Functional Domains

**DOI:** 10.1371/journal.pone.0051479

**Published:** 2012-12-12

**Authors:** Frank G. Oppenheim, Eva J. Helmerhorst, Urs Lendenmann, Gwynneth D. Offner

**Affiliations:** 1 Department of Periodontology and Oral Biology, Boston University Henry M. Goldman School of Dental Medicine, Boston, Massachusetts, United States of America; 2 Department of Medicine, Boston University Medical Center, Boston, Massachusetts, United States of America; Instituto de Salud Carlos III, Spain

## Abstract

The human bodily defense system includes a wide variety of innate antimicrobial proteins. Histatins are small molecular weight proteins produced by the human salivary glands that exhibit antifungal and antibacterial activities. While evolutionarily old salivary proteins such as mucins and proline-rich proteins contain large regions of tandem repeats, relatively young proteins like histatins do not contain such repeated domains. Anticipating that domain duplications have a functional advantage, we genetically engineered variants of histatin 3 with one, two, three, or four copies of the functional domain by PCR and splice overlap. The resulting proteins, designated reHst3 1-mer, reHist3 2-mer, reHis3 3-mer and reHist3 4-mer, exhibited molecular weights of 4,062, 5,919, 7,777, and 9,634 Da, respectively. The biological activities of these constructs were evaluated in fungicidal assays toward *Candida albicans* blastoconidia and germinated cells. The antifungal activities per mole of protein increased concomitantly with the number of functional domains present. This increase, however, was higher than could be anticipated from the molar concentration of functional domains present in the constructs. The demonstrated increase in antifungal activity may provide an evolutionary explanation why such domain multiplication is a frequent event in human salivary proteins.

## Introduction

In the last decades, a number of cationic proteins have been identified which have molecular weights ranging between 2,000 to 6,000 Da and potent antimicrobial activities [1], [2], [3]. Among these are histatins, a family of positively charged proteins secreted by the human parotid and submandibular glands [4], [5]. Histatins exhibit anticandidal activity [5], [6], [7] and may play an important role in the oral cavity to prevent infections with opportunistic fungal pathogens. All three major human histatins, histatin 1, 3, and 5, exhibit killing activity towards *Candida albicans* blastoconidia. The concentrations required for 50% killing (LC_50_) of *C. albicans* blastoconidia are 6.3 µM, 4.2 µM, and 2.0 µM for histatin 1, 3, and 5, respectively [8]. Previously, the antifungal domain of histatins has been localized to a 1,875 Da domain containing 14 amino acid residues in the middle region of histatin 3 [9], [10], [11]. This domain is highly conserved in the three major human histatins, and can also be found in histatins from the subhuman primate *Macaca fascicularis* [12]. It comprises amino acid residues 12–25 of histatin 3 (RKFHEKHHSHRGYR) and exhibits an LC_50_ of 0.3 µM which surpasses the killing activity of all native histatins [8]. The discovery that a fraction of the protein is as active, or even more active, than the entire molecule from which it is derived has important implications. First, on a physiological level, native histatins undergo enzymatic cleavage upon entering the oral cavity [13], [14], [15] which may generate histatin-derived peptides with enhanced biological activity. Second, of clinical importance is the potential exploitation of such functional bioactive peptides for therapeutic purpose [16].

Many proteins in nature contain tandem repeats of individual domains. Examples of salivary proteins containing duplicated domains are the large family of basic proline-rich proteins, also called bPRPs [17], MG1, the large mucous glycoprotein [18] and MG2, the small mucous glycoprotein [19]. In contrast to the latter proteins, histatins are evolutionarily young proteins as evidenced from the fact that they occur exclusively in salivary secretions of man and old world monkeys [20], [21]. From their restricted presence in higher primates and absence from other mammalian species, it can be speculated that the evolutionary time frame has not been adequate to develop histatins with multiple repeats of the active domain. Mimicking such evolutionary trends, we have previously generated a recombinant variant of histatin 3 containing one tandem repeat of a domain comprising amino acids 13–24 and found that duplication of this domain led to a slight increase in antifungal activity [22], [23].

In the present investigation, we tested the hypothesis that functional domain multiplication would amplify the antifungal properties of histatins. PCR and splice overlap extension were used to genetically engineer histatin 3 constructs containing one functional domain (reHst3 1-mer) and variants containing two (reHst3 2-mer), three (reHst3 3-mer), and four (reHst3 4-mer) repeats of the functional domain. The effect of such domain multiplication on antifungal activities towards *C. albicans* blastoconidia and hyphenated cells was determined.

## Materials and Methods

### Strains and Growth Conditions

*Escherichia coli* JM109 (Promega, Madison, WI) was used for cloning, and *E. coli* BL21(DE3) (Novagen Inc, Madison, WI) was used for the expression of recombinant histatin 3 variants. Bacteria were grown on Luria-Bertani (LB) agar plates or in modified LB liquid medium containing ampicillin at concentrations of 50 µg·l^−1^ and 100 µg·l^−1^, respectively. *C.albicans* ATCC44505 was grown on Sabouraud dextrose agar (Difco, Detroit, MI) at 37 °C for 48 hrs.

### PCR Reactions and Amplification of Histatin 3 Variants

The PCR primers used in this study are listed in Table 1 and all reagents were from Invitrogen (Carlsbad, CA). Standard PCR reactions were performed using a Gene Amp PCR system 2400 (Applied Biosystems, Foster City, CA) with an initial denaturation step at 94°C for 4 min followed by 35 cycles at 94°C (30 s), 55°C (30 s), and 68°C (45 s) and finally a 10 min extension at 68°C. Reaction products were analyzed on a 2% agarose gel and DNA fragments were excised and purified using standard procedures.

**Table 1:** PCR primers used in this study.

Primer	DNA Sequences of oligonucleotides^1^
S-1	5′- GGT CGT GGG ATC CCC ATG GAT TCA CAT GCA
S-3	5′- GGT CGT GGG ATC CCC ATG GAT TCA CAT GCA AAG AGA CAT CAT GGG TAT AAA **AGA AAA TTC CAT GAA AAG CAT CAT TCA CAT CGA GGC TAT AGA AGA AAA TTC CAT GAA AAG CAT CAT TCA CAT CGA GGC TAT AGA**
AS-1	5′- ATG CTT TTC ATG GAA TTT TCT TCT ATA GCC TCG ATG
AS-2	5′- TCT ATA GCC TCG ATG TGA ATG ATG CTT TTC ATG GAA TTT TCT TCT ATA GCC
AS-3	5′- ATG CAT GGA TCC CTG AAG ATA TCA ATT GTC
Histatin 3	5′- GAT TCA CAT GCA AAG AGA CAT CAT GGG TAT AAA **AGA AAA TTC CAT GAA AAG CAT CAT TCA CAT CGA GGC TAT AGA** TCA AAT TAT CTG TAT GAC AAT

1 Bolded nucleotide regions represent the functional domain; underlined nucleotide regions represent the BamHI cleavage site.

### Plasmid Construction and Transformation

ReHst3 1-mer, reHst3 2-mer, reHst3 3-mer, and reHst3 4-mer constructs were cleaved with BamHI and ligated into the BamHI site of the pGEX-3X, which is a bacterial GST expression vector (GE Healthcare, Piscataway, NJ). Transformation of the resulting plasmids into JM109 cells was performed according to the manufacturer’s instructions.

### Plasmid Sequencing

PCR screening of transformant colonies was performed with pGEX-3X sense and antisense primers and products were analyzed on a 2% agarose gel. Positive colonies were inoculated into 5 ml of LB medium and plasmids were isolated from cultures grown for 16 hr at 37°C and sequenced in the Department of Genetics and Genomics at Boston University Medical Center to verify in frame ligation of histatin inserts.

### Expression of Recombinant Histatin 3 Variants

To express the recombinant histatin 3 constructs, *E. coli* BL21 (DE3) cells were transformed with each of the pGEX-3X expression plasmids. Single colonies were transferred to 100 ml of LB/amp medium and cultures were grown for 18 hr at 37°C with vigorous shaking. A 1.0 ml aliquot of the liquid culture was transferred into 2 l of LB/amp medium and the culture was incubated as described above until the OD_600_ reached a value of 0.8 to 1.2 absorbance units. Expression of recombinant protein was induced by the addition of IPTG to a final concentration of 0.1 mM. After 3 hr at 37°C, cells were harvested by centrifugation and the pellet was washed twice with ice cold 20 mM Tris-HCl at pH 7.5 and resuspended in lysis buffer containing 20 mM Tris-HCl at pH 7.5, 5 mM MgCl_2_, 10 µg/ml DNase I and 0.25 mg/ml lysozyme. Subsequently, cells were disrupted on ice using a Branson sonicater with 3 bursts of 30 sec each and the lysate was centrifuged at 18,000×g for 20 min at 4°C.

### Membrane Protein Preparation

Preliminary experiments showed that the reHst3 1-mer and reHst3 2-mer were present in the bacterial membrane fraction while the larger constructs were present in inclusion bodies. To isolate the reHst3 1-mer and reHst3 2-mer products, the pellet from the cell lysate was suspended in extraction buffer containing 20 mM Tris-HCl, 1% Triton X-100, 1% deoxycholic acid and 1 mM EDTA, pH 7.5. Membranes were solubilized by sonication to clarity. The solution was dialyzed against deionized water for 16 hr at 4°C and the resulting membrane protein preparation containing the reHst3 1-mer or reHst3 2-mer were lyophilized and stored at –20°C until use. To isolate reHst3 3-mer and reHst3 4-mer from inclusion bodies, the pellet from the cell lysate was suspended in extraction buffer and membranes were dissolved by sonication. Inclusion bodies were recovered by centrifugation at 11,000×g for 20 min at 4°C. The pellet containing the inclusion bodies was suspended in 20 mM Tris-HCl, containing 1 mM DTT and 6 M urea and solubilized by sonication. The solution was dialyzed for 16 hr against deionized water resulting in the precipitation of inclusion bodies which were subsequently harvested by centrifugation at 11,000×g for 20 min at 4°C. Pellets were washed with deionized water, centrifuged again and stored frozen until further use.

### Cyanogen Bromide Cleavage

Fusion proteins were cleaved with CNBr at a methionine residue introduced between the GST fusion partner and the first amino acid of the recombinant histatin variants. Inclusion body preparations or lyophilized membrane protein preparations were dissolved in a minimal amount of 70% formic acid in a glass vial. The reaction mixture was agitated on a magnetic stirrer, solid CNBr was added to a final concentration of approximately 10 mg ml^−1^, and the vial was purged with nitrogen. After 21 hr of incubation, reactions were diluted 3-fold with water and the HCN formed was purged with nitrogen. The solutions containing the CNBr cleavage products were frozen and lyophilized.

### RP-HPLC-Purification

CNBr digests were resuspended in a solution containing 4 M urea and 0.1% trifluoroacetic acid. Samples were centrifuged for 20 min at 25,000×g, dialyzed, passed through a filter with a pore size of 0.45 µM and subjected to reversed-phase HPLC using a preparative C-18 column (2.15×30 cm; ODS 120T, Tosohaas, Montgomeryville, PA). Proteins were eluted at a flow rate of 5 ml min^−1^ with a gradient of eluent A (H_2_O, trifluoroacetic acid 0.1%), and eluent B (80% acetonitrile, 20% H_2_O trifluoroacetic acid 0.1%). The proportion of eluent B was linearly increased from 10% to 40% over a time period of 80 min. For each purification, fractions containing the recombinant histatin 3-mer of interest were combined, concentrated to approximately 30% of the original volume by flash evaporation (Rotavapor M, Büchi Labortechnik AG, Flawil, Switzerland) and lyophilized. All fractions were dissolved in 5 ml of water containing TFA (0.1%) and further purified on the same column by isocratic elution using the appropriate acetonitrile concentration containing 0.1% TFA. Fractions containing purified histatin 3 variants were pooled, concentrated by flash evaporation and lyophilized.

### Protein Concentration Determination

The protein concentrations in stock solutions of recombinant histatins in water were determined by measuring the absorbance at 280 nm (1). Molar extinction coefficients (ε) of 5120, 6400, 7680 and 8960 (M^−1^.cm^−1^) were used for the reHst3 1-mer, 2-mer, -3-mer and 4-mer, respectively.

### Killing Assays

For blastoconidia killing assays *C. albicans* cells were grown at 30°C in ¼ strength Sabouraud dextrose broth (Difco). Partial strength (¼ diluted) broth was used since fungal growth in this medium shows the typical lag-, log- and stationary- phases, whereas in undiluted broth the point at which the stationary phase was reached was less discernible (unpublished observations). A sample of exponentially growing cells was diluted in 10 mM potassium phosphate buffer (PPB), pH 7.4, to give a cell density of approximately 10^5^ cells ml^−1^. Aliquots of 50 µl of this suspension were added to the wells of a 96-well microtiter plate and cells were allowed to attach for 15 min. Subsequently, 50 µl of the histatin solutions (in 10 mM PPB) were added to the wells and the microtiter plate was incubated for 60 min at 37°C. After incubation, wells were washed with 10 mM PPB and 100 µl of molten Sabouraud's dextrose broth containing 2% agarose (45°C) was added to each well. After 5–6 hr incubation at 30°C, surviving cells had divided and formed colonies whereas dead cells remained as single cells. Microcolonies, defined as a cluster of more than five contiguous cells, and single cells were counted to determine cell viability as described previously [8].

For germ tube killing assays, *C. albicans* cells were grown for 36 hr on Sabouraud dextrose agar, suspended in 10 ml RPMI-1640 medium buffered with 10 mM HEPES to pH 7.4, and diluted in the same medium to a concentration of 10^5^ cells ml^−1^. Aliquots of 50 µl of this suspension were added to the wells of a microtiter plate and incubated for 3 hr at 37°C. These conditions typically converted over 95% of blastoconidia into germinated cells. Subsequently, plates containing the attached germinated cells were washed twice with 10 mM PPB, and 50 µl of the recombinant protein solutions (in 10 mM PPB) were added. Plates were incubated at 37°C for 10, 30, 60, 90 or 120 min and overlayed with molten Sabouraud's dextrose broth (45°C) containing 2% agarose. After 6–8 hr of incubation at 30°C surviving germinated cells growing in micro colonies and single dead cells were counted to determine the percentage of cell survival.

### Statistical Analysis

Statistical differences in LC_50_ values between blastoconidia and germ tubes treated with reHst3 1-mer, 2-mer, 3-mer, or 4-mer were investigated with the Friedman test (1-sided), with a level of significance set at p<0.05, using SPSS v20.0 software.

## Results

### Generation of Histatin Multimers

**Figure 1:**
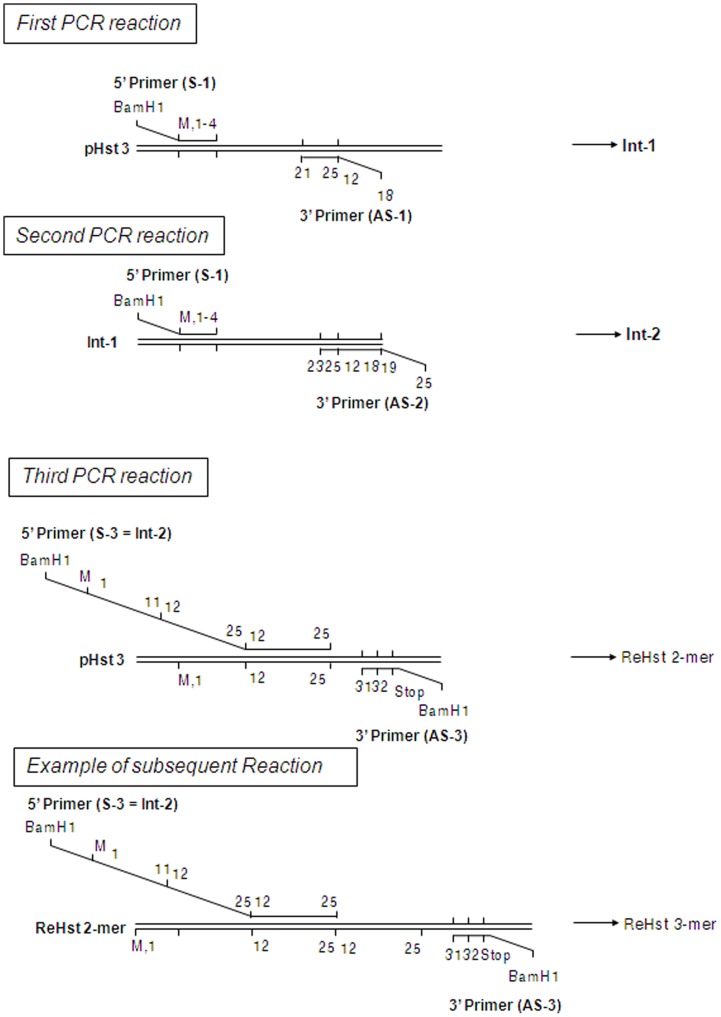
PCR reactions using splice overlap extension to generate recombinant histatin variants. In the first PCR reaction primer S-1, anti-sense primer AS-1, and template pHst3 were used to generate an intermediate product designated Int-1. In the second PCR reaction, Int-1 served as the template with S-1 and AS-2 as primers to generate a second intermediate product (Int-2). Int-2 was used as the sense primer (S-3) for the following reaction with AS-3 as the anti-sense primer and pHst3 as the template to generate the reHst3 2-mer construct. In subsequent cycles, Int-2 annealed with any of the functional domains of the products from preceding cycles to generate constructs with multiple functional domains.

**Figure 2:**
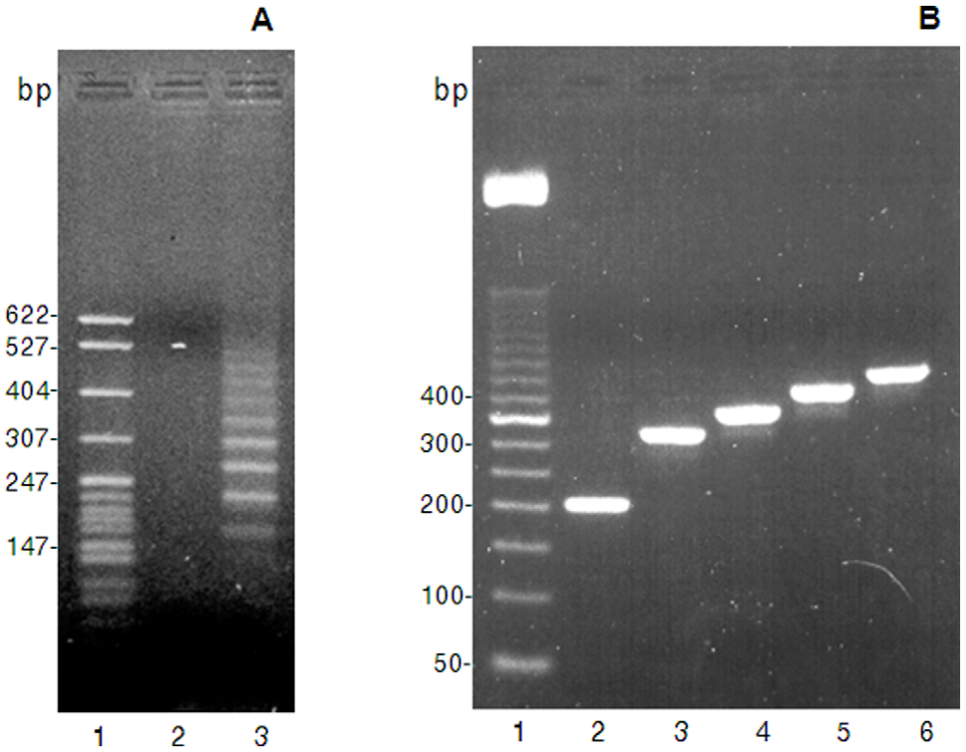
Analysis of recombinant histatin 3 constucts containing multiple functional domains on a 2% agarose gel. A, lane 1: pBR22-Hsp I marker; lane 2: empty; lane 3∶16.5 µl of the final PCR reaction products. B, Analysis of inserts in histatin 3 constructs in pGEX-3X. Inserts were amplified using vector specific primers and an aliquot (1 µl) of each PCR reaction was analyzed on a 2% agarose gel. Lane 1, Gibco BRL 50 bp DNA ladder (6 µg); Lanes 2–6, PCR products of pGEX-3x, pHst3 1-mer, pHst3 2-mer, pHst3 3-mer, and pHst3 4-mer, respectively.

DNA fragments encoding recombinant histatin 3 with one, two, three, or four functional domains were generated in sequential PCR reactions using overlap extension [24]. Each construct contained a BamHI restriction site, a start codon, the N-terminus of histatin 3, one or more functional domains, and the C-terminal 7 residues of native histatin 3. A recombinant fragment containing one functional domain was obtained using primers S-1 and AS-3 and a previously isolated histatin 3 expression plasmid (pHst3) as template (data not shown). Three sequential PCR reactions were carried out to prepare recombinant histatin variants with two, three or four functional domains (Fig. 1). First, using the pHst-3 template as described above, the sense primer S-1 and the antisense primer AS-1 amplified a fragment encoding residues 1–25 of histatin 3 and half of a second functional domain (amino acid residues 12–18; Fig. 1). This intermediate fragment (Int-1) was used as a template in the second PCR reaction with primers S-1 and AS-2, yielding a 135 bp intermediate product (Int-2) with two tandem repeats of the functional domains. The intermediate product (Int-2) was used as the sense primer (S-3) in the last PCR reaction with antisense primer AS-3 and pHst3 as the template. The S-3 primer annealed in cycle 1 to DNA encoding amino acids 12–25 of the original histatin 3. This resulted in a recombinant histatin 3 fragment with two functional domains (reHst3 2-mer). In cycle 2 to 35, the primer S-3 annealed either with the original template or with the product of the first cycle. In the latter case a gene product encoding histatin 3 with either two or three functional domains was obtained depending on whether S-3 annealed to the second or the first functional domain of the reHst3 2-mer, respectively. In the third cycle the S-3 primer annealed with the original template and the fragments generated in the first and the second cycles. Consequently, histatin 3 with two, three and four functional domains were obtained from this reaction. Theoretically, with every subsequent cycle a product containing one additional functional domain could be generated. Indeed, fragments with sizes corresponding to up to 10 functional domains could be observed after the final PCR reaction (Fig. 2A). Adequate amounts of DNA for further studies were obtained from PCR fragments containing 1 to 4 functional domains.

After ligation of the recombinant histatin 3 fragments into pGEX-3X, the sizes of the inserts were verified by PCR using vector- specific primers. As expected, fragments of 201 bp, 312 bp, 354 bp, 396 bp, and 438 bp were obtained including 3 bp for methionine and 12 non-translated bp, and correspond to vector alone and histatin 3 constructs with 1, 2, 3 and 4 functional domains, respectively (Fig. 2B). Sequence analysis of inserts in these constructs revealed that all histatin 3 fragments were cloned in frame into the pGEX-3X vector and the deduced protein sequences of the four constructs are given in Table 2. Interestingly, some of the colonies examined contained histatin 3 constructs (ReHst3 2-mer, ReHst3 3-mer and ReHst3 4-mer) in which two point mutations were detected, a G->C silent mutation in the third base of the glycine codon at position 9 and an A->G missense mutation in the second base of the codon at position 11, leading to replacement of lysine with arginine. Analysis of additional colonies identified ReHst3 3-mer and ReHst3 4-mer constructs containing inserts with the expected sequence (Table 2). However, despite repeated attempts, no colonies containing the expected ReHst3 2-mer sequence were found. Thus, the ReHst3 2-mer used in this study contains an arginine at position 11 (Table 2). Because this mutation was not in the functional domain and did not change the charge of the recombinant histatin compared to the theoretically expected sequence, the strain was still used for further experiments. Due to multiplication of the middle portion of histatin 3, which is enriched in lysine, arginine and histidine residues, the average charge per amino acid residue increased as well as the isoelectric point of the protein in concordance with the addition of functional domains (Table 3).

**Table 2:** Amino acid sequences of recombinant histatin 3 multimers^1^.

Residue	110203040506070
ReHst3 1-mer	DSHAKRHHGYKRKFHEKHHSHRGYRSNYLYDN
ReHst3 2-mer^2^	DSHAKRHHGY**R** RKFHEKHHSHRGYRRKFHEKHHSHRGYRSNYLYDN
ReHst3 3-mer	DSHAKRHHGYKRKFHEKHHSHRGYRRKFHEKHHSHRGYRRKFHEKHHSHRGYRSNYLYDN
ReHst3 4-mer	DSHAKRHHGYKRKFHEKHHSHRGYRRKFHEKHHSHRGYRRKFHEKHHSHRGYRRKFHEKHHSHRGYRSNYLYDN

1 The fungicidal domain sequences are underlined.

2 The bolded R residue in the ReHst3 2-mer indicates an unintentional amino acid substitution introduced by the DNA polymerase.

**Table 3:** Some physico-chemical properties of histatin constructs with multiple functional domains.

Peptide variant	Vector	Number of residues	Molecular Weight (Da)	IEP^1^	charge at pH 7.2	Average chargeper residue	Lysyl residues	Arginyl residues	Histidyl residues
reHst3 1-mer	pHst3-1	32	4062	10	+5	+0.156	4	4	7
reHst3 2-mer^2^	pHst3-2	46	5919	10.4	+9	+0.195	5	8	11
reHst3 3-mer	pHst3-3	60	7777	10.7	+13	+0.217	8	10	15
reHs3 4-mer	pHst3-4	74	9634	10.9	+17	+0.230	10	13	19

1 IEP, isoelectric point.

2 All properties of the reHst3 2-mer were calculated taking into account the Lys to Arg substitution at position 11.

**Figure 3:**
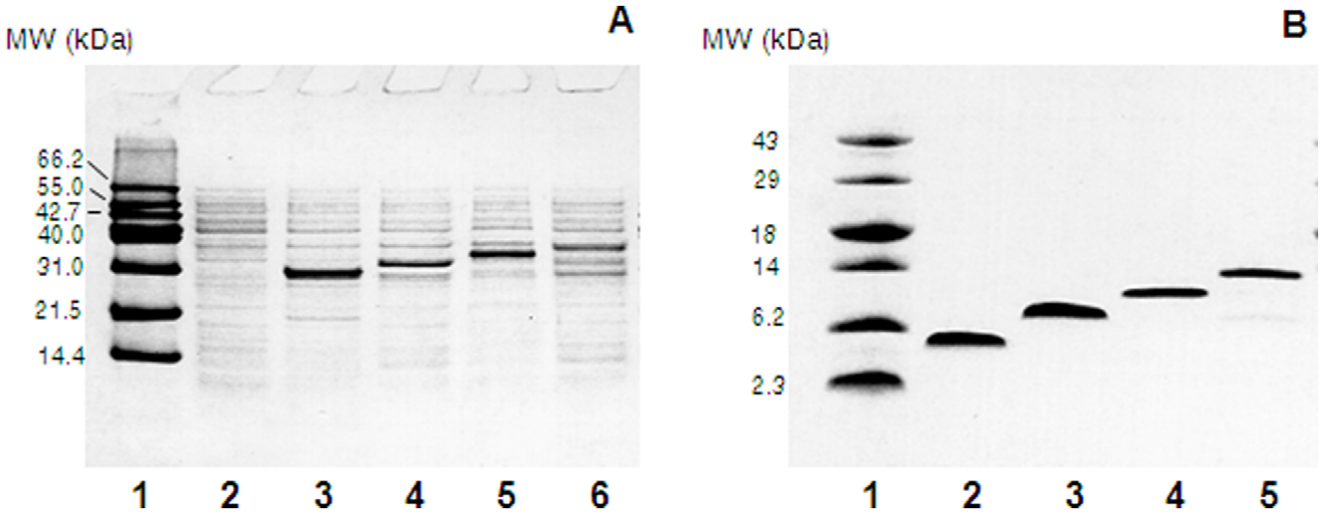
Expression of recombinant glutathion-S-transferse-histatin3 fusion proteins in *E. coli* BL21(DE3) using pGEX-3X as expression vector. A, Cell free extracts were analyzed on a 16.5% Tris-tricine SDS-PAGE. Lane 1, molecular weight marker; lane 2, pGEX-3x/His3-1 before induction; lane 3, induced pGEX-3x/His3-1; lane 4, pGEX-3x/His3-2; lane 5, pGEX-3x/His3-3; lane 6, pGEX-3x/His3-4. B, Tris-tricine SDS-PAGE of purified recombinant histatins. Lane 1, molecular weight marker; lane 2, reHst3 1-mer; lane 3, reHst3 2-mer; lane 4, reHst3 3-mer; lane 5, reHst3 4-mer.

Because the pGEX-3X vector expresses recombinant proteins as fusion proteins with a 26 kDa glutathione-S-transferase (GST) fusion partner, prominent protein bands of approximately 31, 33, 35, or 38 kDa were observed after IPTG induction of cells expressing the reHst3 1-mer, 2-mer, 3-mer, or 4-mer, respectively (Fig. 3A). After cleavage of the recombinant proteins with cyanogen bromide, several other small peptide fragments were produced due to methionine residues in glutatione-S-transferase (data not shown). Therefore, the constructs were purified by preparative RP- HPLC and analyzed by SDS-PAGE. This procedure resulted in pure preparations of recombinant histatin 3 variants with one, two, three, and four functional domains (Fig. 3B). Typically, preparations from 2 liters of bacterial culture yielded 3–10 mg of purified recombinant histatin 3-multimers.

### Evaluation of Candidacidal Activities

**Figure 4:**
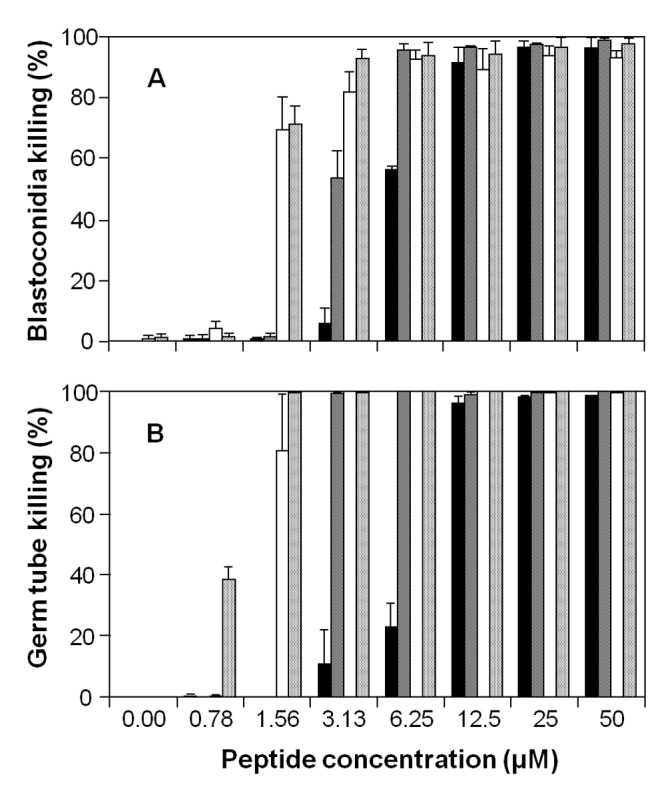
Killing activity of recombinant histatin 3 mers. A, *C. albicans* blastoconidia killing. Cells were grown to exponential phase in ¼ strength Sabouraud dextrose broth; B, Germ tube killing. Formation of germ tubes was induced by incubating blastoconidia in RPMI-1640 for 3 hr at 37°C. Killing was determined for peptide concentrations between 0 and 50 µM after an incubation time of 60 min at 37°C. Black bars, reHst3 1-mer; crosshatched bars, reHst3 2-mer; white bars, reHst3 3-mer; dotted bars, reHst3 4-mer. The experiments were performed in triplicate. The average and standard deviation (SD) of a representative experiment is shown.

**Figure 5:**
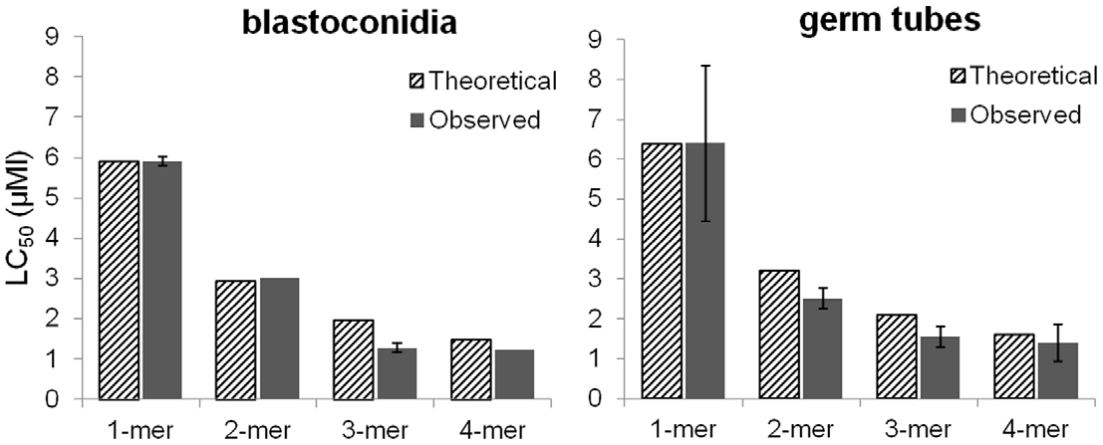
Theoretical and observed LC_50_ values of histatin 3 mers towards *C. albicans* blastoconidia and germ tubes. From three independent histatin dose-effect curves, the average LC_50_ values and standard deviations (SD) were determined (solid bars). The theoretical LC_50_ values were calculated by dividing the observed LC_50_ value for reHst3 1-mer by 2, 3, and 4, respectively (hatched bars).

**Figure 6:**
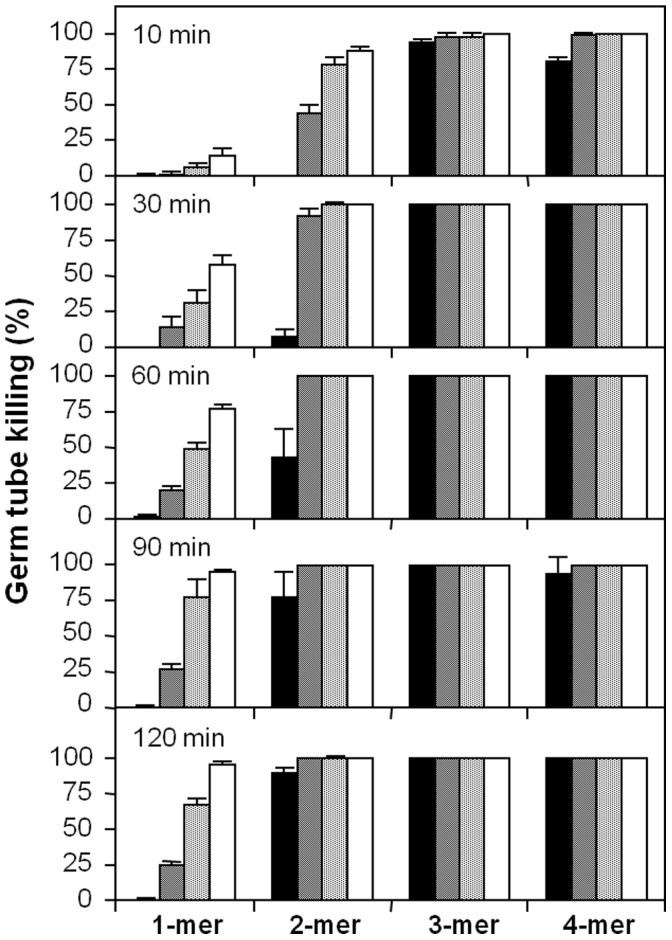
Time dependent killing activity of recombinant histatin 3 variants. Germ tubes were incubated with histatin 3 variants for 10, 30, 60, 90 and 120 min. Peptide concentrations of 1.56 µM (black bars), 3.13 µM (crosshatched bars), 6.25 µM (dotted bars) and 12.5 µM (white bars) were employed. Experiments were conducted in duplicate and the average and deviation from the mean are shown.

For the antifungal susceptibility testing of the various histatin constructs we employed a microcolony assay [8]. The microcolony assay methodology has recently been validated by comparison to Etest and EUCAST standardized antifungal assays [25], [26]. The killing activity of recombinant histatin 3 and variants was tested against *C. albicans* blastoconidia and germ tubes. In both killing assays, native salivary histatin 3 and the recombinant peptide reHst3 1-mer exhibited similar killing activities showing that the recombinant protein expressed in *E. coli* is functionally indistinguishable from histatin 3 isolated from salivary secretion (data not shown). For the blastoconidia cell killing assay, cells were cultured to the exponential growth phase in liquid medium. All four recombinant histatin variants exhibited maximum killing activity towards *C. albicans* blastoconidia, i.e., 98–100% killing, at peptide concentrations of 12.5 µM to 50 µM (Fig. 4A). However, 6.25 µM reHst3 1-mer with one functional domain killed approximately 55% of the cells, while the reHst3 2-mer, 3-mer and 4-mer still exhibited maximum killing activity. At a concentration of 3.13 µM, reHst3 1-mer failed to exhibit significant killing activity while reHst3 2-mer killed 50% of the cells and reHst3 3-mer and reHst3 4-mer killed over 80% at this concentration. At a lower peptide concentration (1.56 µM) only reHst3 3-mer and reHst3 4-mer exhibited killing activity. The LC_50_ values that could be derived from multiple repeats of these experiments were 6.8±0.4, 2.6±0.7, 1.0±0.1 and 1.1±0.1 µM for ReHst3 1-mer, 2-mer, 3-mer, and 4-mer, respectively. Similar differences in antifungal potency among the four histatin variants were observed toward germinated *C. albicans* cells (Fig. 4B). Dose-response curves for germ tube killing were much steeper than those found for blastoconidia. This is illustrated in particular by the abrupt loss in killing activity of reHst3 2-mer between 3.13 and 1.56 µM. Similarly, killing activity dropped from 80% to 0% for reHst3 3-mer between 1.56 and 0.78 µM. From these results the LC_50_ values could be calculated (Figure 5; solid bars). Statistical analysis of the differences between groups indicated a significant decrease in LC_50_ value with an increasing number of copies of the active domain, for the blastoconidia (p = 0.016) as well as the germ tubes (p = 0.025). We also calculated the theoretical LC_50_ values for the multimeric variants by dividing the LC_50_ value for the monomer by 2, 3 and 4, respectively (Figure 5; hatched bars). A comparison of the calculated with the observed LC_50_ values shows a trend that the observed values are lower, suggesting a synergistic effect upon active domain multiplication. To further characterize the enhanced activities of histatin 3 variants, germinated cells were exposed for various time intervals to four different protein concentrations (1.56, 3.13, 6.25 and 12.5 µM). The results show that with an increased number of functional domains, histatin 3 variants killed *C. albicans* cells significantly faster (Fig. 6). For example, the reHst3 3-mer and 4-mer caused cell death within 10 min of incubation (at all concentrations tested) whereas after the same incubation period, >90% of cells exposed to the reHst3 1-mer (at all concentrations tested) survived. Thus it appears that the histatin 3 multimers are not only more active on a molar base, but also kill cells more rapidly than the histatin 3 monomer as the number of copies of the functional domain increases.

## Discussion

We genetically engineered histatin 3 constructs in which the antifungal domain was present in 1, 2, 3, or 4 copies. For the evaluation of the antifungal activity of the variants, we chose to use an assay in which fungal cells are exposed to the antifungal proteins in the adhered form, a condition more representative of the *in vivo* situation than cells in suspension. Assays were conducted in low ionic strength buffers, since histatin activity is salt-sensitive [8], [9], and saliva fluid is hypotonic in nature [27]. The results indicated that, on a molar basis, the multimeric variants were significantly more active than the naturally occurring histatin 3 protein containing only a single copy of the active domain. This was true both for *C. albicans* blastoconidia and germ tubes. The increase in antifungal activity tended to be higher than could be expected from multiplying the activities of the monomer, demonstrating the functional advantage of active domain multiplication.

**Figure 7:**
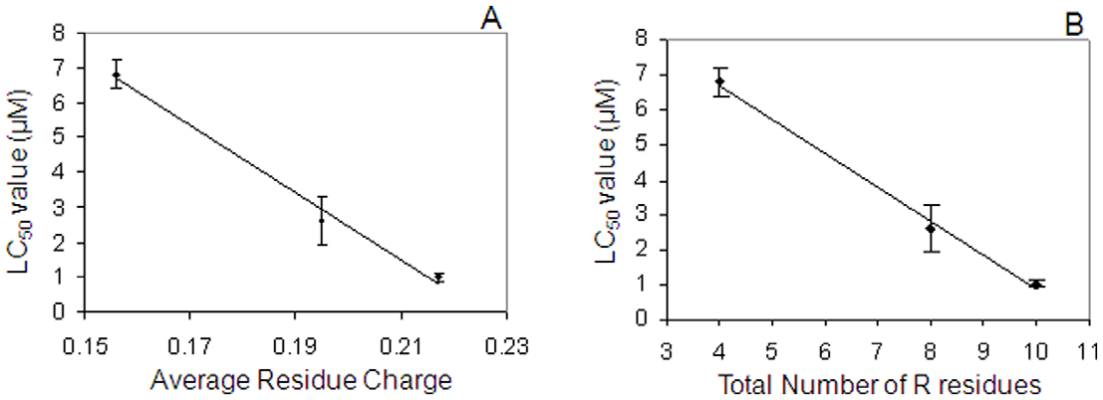
Protein properties affecting the antifungal activity of the ReHst constructs. Relationship between the average residue charge (A) or the number of arginine residues (B) and the LC_50_ values.

It is tempting to speculate on the essential protein characteristics which bestow histatins with antifungal activities, and on the structural basis for the enhanced activities of the multimers. The histatin variants investigated differ from each other not only with respect to the number of residues, but also in IEP, charge at neutral pH, average charge per residue, and the number of positively charged residues (Table 3). The net increase of positively charged amino acids per functional domain is five due to the presence of 2 lysine and 3 arginine residues. Therefore, multiplication of the functional domain not only yields larger but also more cationic macromolecules. Furthermore, addition of each domain adds 4 histidine residues to the primary structure. To understand how multiplication of the lysine, arginine and histidine-rich functional domain enhances antifungal activity, it is worthwhile to consider the role of these amino acid residues in cell killing. It has been demonstrated that replacement of histidyl residues in the active domain of histatin 5 with glycine reduces antifungal activity [28], while replacement of the histidines with lysines increased activity [9]. Furthermore, substitution of positively-charged lysyl and arginyl residues of the functional domain in histatin 5 with glycine reduced killing activity [28]. These data point toward an important role of positively charged residues and average charge per residue for antifungal activities, which is consistent with the higher antifungal activities observed for the more cationic multimeric histatin constructs. Interestingly, the number of arginine residues as well as the average residue charge of the ReHst3 1-mer, 2-mer and 3-mer show almost perfect inverse linear correlations with the LC_50_ values (R^2^ values of 0.996 and 0.991, respectively; Fig. 7) suggesting that these parameters in particular may be mechanistically related to function. It should be noted however that the LC_50_ value of the ReHst3 4-mer was almost identical to that of the 3-mer, suggesting that the maximal gain in functional efficacy is reached upon threefold multiplication of the active domain. This limiting benefit in function may be related to size-limitations with respect to the cellular uptake mechanism of histatins [29], [30].

There is a clear need for new antifungal drugs given the increase in fungal resistance to commonly applied antimycotics. In general, the industrial focus is on generating small molecules or peptides for clinical applications, since these can be synthesized on a large scale and at acceptable production costs. For any new antifungal drug, additional testing would be required to establish the suitability for clinical exploitation. Such tests include toxicity towards mammalian cells, assessment of their activity towards a larger number of fungi including clinical isolates and strains that developed resistance to other antifungal drugs. For native histatins experimental evidence for clinically relevant activities has been obtained in several studies [31], [32], [33], [34], [35], and a histatin-derived peptide, P-113, has reached clinical trials [36]. Toxicity tests would have to be performed on the multimeric histatin constructs as well, if these were developed for therapeutic purposes. However, the primary focus of this study was not to generate a new drug, but to investigate the biological advantage of domain multiplication which was demonstrated successfully.

Overall, our data support the contention that several fold multiplication of active domains can yield proteins with higher antifungal activities on a molar basis. This finding may provide for the first time an evolutionary explanation why such domain multiplication is a frequent event in human salivary proteins.
